# Tumor M2-PK: A novel urine marker of bladder cancer

**DOI:** 10.1371/journal.pone.0218737

**Published:** 2019-06-27

**Authors:** Weiya Liu, Benjamin L. Woolbright, Karim Pirani, Ryan Didde, Erika Abbott, Gaurav Kaushik, Paige Martin, Jill Hamilton-Reeves, John A. Taylor, Jeffrey M. Holzbeierlein, Shrikant Anant, Eugene K. Lee

**Affiliations:** 1 Department of Urology, University of Kansas Medical Center, Kansas City, Kansas, United States of America; 2 Department of Surgery, University of Kansas Medical Center, Kansas City, Kansas, United States of America; University of Central Florida, UNITED STATES

## Abstract

**Purpose:**

Bladder cancer is a “Warburg-like” tumor characterized by a reliance on aerobic glycolysis and expression of pyruvate kinase M2 (PKM2). PKM2 oscillates between an active tetramer and an inactive dimer. We aim to further characterize PKM2, in particular PKM2 dimer, as a urinary biomarker of bladder cancer and a potential target for treatment.

**Methods:**

HTB-9, HTB-5, and UM-UC3 bladder cancer cells were assessed for proliferation under differential glucose levels using the hexosaminidase assay. Western blot and Blue-native analysis was performed for protein expression of PKM2. Shikonin, an herb that is known to bind and inhibit PKM2, was utilized to determine if PKM2 has a role in glucose usage and cellular proliferation in bladder cancer cells by caspase activity assay. Institutional review board approval was obtained to collect healthy control and bladder cancer patient urine samples. The ScheBo M2-PK EDTA Plasma Test was performed on urine samples to assess urine Tumor M2-PK values.

**Results:**

The three bladder cancer cell lines tested all demonstrate statistically significant increases in proliferation when exposed to higher level of glucose (200mg/dL). Similarly, low doses of glucose (25mg/dL) result in reduced proliferation. Increased cell growth in higher glucose concentration correlated with up-regulation of PKM2 protein expression. Shikonin, a PKM2 inhibitor, reduced cell proliferation and switched PKM2 isoforms from the dimer to tetramer. Lastly, dimer PKM2 (Tumor-M2PK) levels were assessed in the urine samples from bladder cancer (Bca) patients and healthy controls. Tumor M2-PK significantly correlated with the presence of BCa in our subjects.

**Conclusions:**

Our studies demonstrate the potential of PKM2, specifically the dimer (Tumor-M2PK) as a target of drug therapy and as a urinary marker for bladder cancer.

## Introduction

Many malignancies, including bladder cancer, have a heightened affinity for glucose and demonstrate a shift toward an aerobic glycolysis-dependent metabolism, commonly referred to as the Warburg effect [1, 2]. Aggressive bladder cancer cell lines increase pyruvate consumption for glycolysis and increase lactate production despite the availability of sufficient oxygen, another hallmark of this phenomenon [3]. One of the principal drivers of the Warburg effect is pyruvate kinase (PKM), the rate-limiting last step in the glycolytic pathway, which catalyzes the reaction of phosphoenolpyruvate (PEP) and ADP to pyruvate and ATP [4]. Clinically, overexpression of PKM2 is associated with tumor size, nodal metastatic disease, stage, disease progression, and overall survival in squamous cell carcinoma [5, 6]. PKM1 and PKM2 are encoded by the PKM gene, and are different splicing products of the M-gene (exon 9 for PKM1 and exon 10 for PKM2). The PKM1 isozyme is expressed in organs that are strongly dependent upon a high rate of energy regeneration, such as muscle and brain. Switching from PKM2 to PKM1 subtype results in decreased lactate production, increased oxygen consumption, and reduced ability to form lung tumor in mouse xenografts [7].

While the presence of PKM isoform switching has been questioned in other tumors, bladder is one of the few tissues where there is a confirmed increase in the PKM2:PKM1 ratio between cancerous and normal tissue as normal bladder expresses high levels of PKM1 [8]. Additionally, in bladder cancer, PKM2 expression by immunohistochemistry correlates with increasing grade in human tumor samples when compared to normal urothelium [9]. Furthermore, Shikonin, a Chinese herbal supplement, is found to inhibit PKM2, and increase the sensitivity of cisplatin in bladder cancer cell lines [10]. Lastly, when examining data from the Tumor Genome Cancer Atlas registry (TCGA) for bladder cancer specimens, RNA sequencing data demonstrate that 97% of the 131 invasive tumors expressed transcripts of PKM2 versus only 3% for PKM1 [11]. These results suggest an important role of PKM2 in invasive bladder cancer tumors.

PKM2 has two different forms and is dynamic in cellular activity [12]. The tetrameric form of PKM2 has a high affinity to its substrate phosphoenolpyruvate (PEP), and is highly active at physiological PEP concentrations, which is the case in differentiated tissues and most normal proliferating cells. The dimeric form of PKM2 is characterized by a low affinity to its substrate PEP and is nearly inactive at physiological PEP concentrations, which is the case in tumor cells. PKM2 oscillates between an active tetramer and an inactive dimer, and the dimerization of PKM2 in tumor cells is induced by direct interaction of PKM2 with different oncoproteins [12]. Counterintuitively, it is the inactive dimer form of PKM2 that is predominant in cancer cells [13, 14]. The inactivity of dimer PKM2 halts the progression of glycolysis and allows for precursor substrates to accumulate and be diverted to nucleic acid, amino acid, and phospholipid synthesis, all critical for cellular growth and proliferation [12]. We hypothesize that the dimer form of PKM2, also known as Tumor M2-PK, would be elevated in bladder cancer, and the inhibition of PKM2 would reduce bladder cancer cell growth. In this study, we further characterized PKM2 in bladder cancer cell lines and establish urinary Tumor M2-PK as a potential urine based biomarker for bladder cancer.

## Materials and methods

### Materials and cell culture

Authenticated human high-grade bladder cancer cell lines HTB-9, HTB-5, and UM-UC3 were obtained from ATCC (ATCC–Manassas, VA). The bladder cancer cells were cultured in DMEM media (10-014-CV, Corning, Manassas, VA) with 10% FBS, 1% penicillin/streptomycin (100 IU/ml/100 mg/ml), 2% MEM vitamins, and 1% MEM non-essential amino acids. All cells were maintained at 37°C with 5% CO_2_. Freeze-down stocks of the original characterized cell lines were cryopreserved in liquid nitrogen. All experiments were performed using cells with < 20 passages and < 3 months in continuous culture. PKM2 antibody was purchased from Cell Signaling Technology (Danvers, MA). Shikonin was acquired at United States Pharmacopeia grade via Sigma-Aldrich. All chemicals were acquired from Sigma-Aldrich unless otherwise noted.

### Cell proliferation analysis

Cell proliferation was measured by hexosaminidase assay as previously described [15]. Briefly, HTB-9, HTB-5 and UM-UC3 cells were plated at 1,000 cells per well in 96 well plates and allowed to adhere overnight. Media with different glucose concentrations was changed every 24 hours, for 7 days. During measurement, the media was removed and hexosaminidase substrate solution in citrate buffer was added and the absorbance was measured at 405 nm. Experiments were conducted in quadruplicate and independently repeated at least three times. Analyzed data are presented as average ± standard error of mean.

### Western blot analysis

HTB-9, HTB-5, and UM-UC3 cells were harvested in cold PBS and lysed with RIPA buffer: 50mM Tris-HCl pH 7.5, 150mM NaCl, containing 0.1% SDS, 1% Igepal, 1% sodium deoxycholate, protease and phosphatase inhibitor cocktail (Sigma-Aldrich, Inc., St. Louis, MO) by three freeze-thaw cycles using liquid nitrogen and a 37°C water bath. Protein concentration was determined using DC Protein Assay (Bio-Rad Laboratories, Hercules, CA). Equal amounts of protein (20 μg) were loaded on a Novex E-PAGETM 8% protein gel (Life Technologies), transferred to a nitrocellulose membrane by Novex iBlot Gel Transfer system (Invitrogen, Carlsbad, CA), blocked in TBS-T containing 5% milk, and probed with primary antibodies (1:1000 dilution). Membranes were incubated with a horseradish peroxidase-conjugated secondary antibody, developed and visualized with Li-COR Odyssey Image system. All Western blots were probed for the loading control β-actin. The data were representative of at least three independent experiments (*n* = 3).

### Caspase activity assay

HTB-9, HTB-5, and UM-UC3 cells were plated at 5.0 × 10^5^ cells per well in 6-well dishes and allowed to adhere overnight. Shikonin was dissolved in media at 10 μM, and allowed to incubate with cells for 48 hours. Caspase activity was assessed by measuring the amount of relative fluorescence units generated over 30 minutes per mg of protein using a fluorescent substrate (Ac-DEVD-AMC) as described previously.

### Blue-Native gel electrophoresis

After 48h of Shikonin treatment, BN lysates were prepared from UM-UC3 cells in 20 mM Bis-Tris (pH7.4), 125 mM caproic acid, 20 mM KCl, 2mM EDTA, 5 mM MgCl_2_, 10% glycerol and 2% n-dodecyl beta-D-maltoside (DDM) followed by three freezing and thawing cycles and centrifugation at 14,000 g for 30 min at 4°C. Protein concentration was determined as described above and equal amounts of protein loaded on a Native PAGE Novex 3–12% Bis-tris gel (Invitrogen, Carlsbad, CA) and electrophoresed according to manufacturer’s instructions.

### Patient selection and ethics statement

This study was approved by University of Kansas Medical Center under IRB #141546 and IRB #140279. Patient urine samples were acquired with written consents and in agreement with the Helsinki Accords on human tissue collection, no minors consented. 50 urine samples were from patients with known bladder cancer, and 10 normal control urine samples were obtained from volunteers within the Department of Urology.

### Urine tumor M2-PK analysis

Tumor M2-PK analysis was performed using the ScheBo M2-PK EDTA Plasma Test (ScheBo Biotech, 35394 Giessen Germany). After optimization of the protocol with dilution factors, urine samples were evaluated using the manufacturer’s suggested protocol. These results were performed independently three times.

### Statistics

All data are mean ± SEM. ANOVA was used to assess differences between groups using Dunnett’s post-hoc to compare to controls or Student Neuman Keuls for differences between groups. p<0.05 was considered significant.

## Results

### Glucose enhances bladder cancer cell proliferation

**Fig 1:**
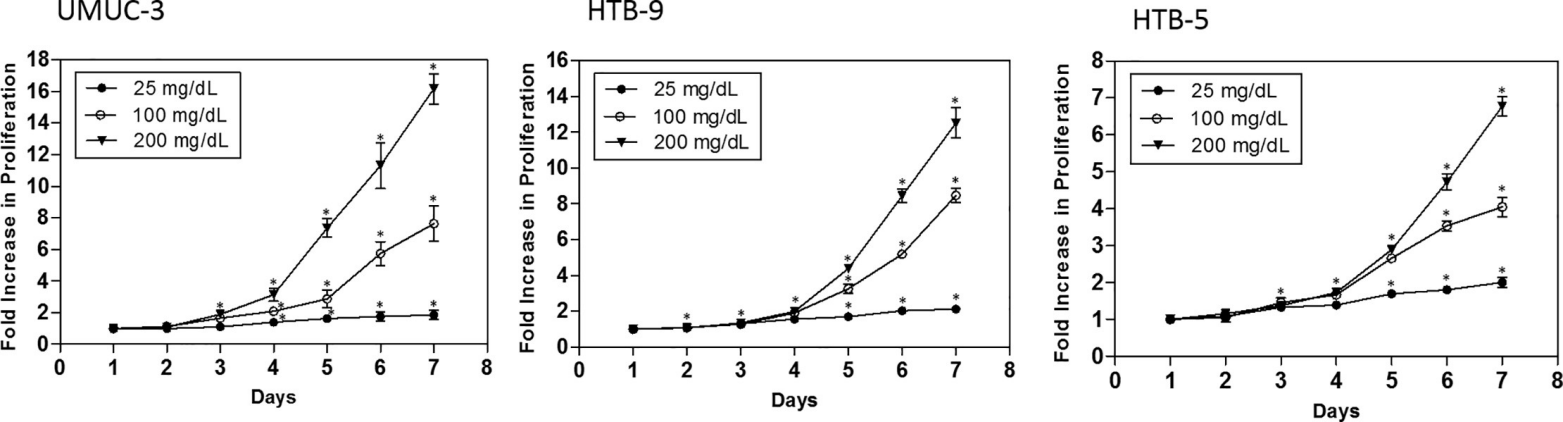
Glucose enhances BCa cell proliferation. Bladder cancer cells were seeded at 1000 cells/well in 96-well plates. Cells were exposed to different glucose concentrations (25 mg/dL, 100 mg/dL, and 200 mg/dL) for 7 days. Hexosiminidase assay was performed every 24 hours. Data were analyzed from four independent experiments, each data point represents the mean ± S.E.M. * indicates significant two-tailed t-test *p* value of < 0.05, compared with Day.1.

Bladder cancers have been demonstrated to be highly glycolytic tumors [1]. We sought to confirm the dependence of bladder cancer cells on glucose levels for proliferation and the role PKM2 plays in this process. To confirm this *in vitro*, HTB-9, HTB-5 and UM-UC3 bladder cancer cells were exposed to three differing concentrations of glucose (25 mg/dL, 100 mg/dL, and 200 mg/dL) and cell proliferation was assessed. Normal blood glucose levels in human, being approximately 100 mg/dL was selected, with 25 mg/dL and 200 mg/dL representing lower and higher concentrations. Bladder cancer cells were cultured for 7 days, with media refreshed every 24 hours to maintain a consistent glucose level. All three bladder cancer cells in the 200 mg/dL glucose concentration demonstrated increase in proliferation over the 100 mg/dL condition, for HTB-9 cells, the significant increase (*p* value of < 0.05) started to show at Day 2, and for HTB-5 and UM-UC3 cells, the significant difference in proliferation started to show at Day 3 (**Fig 1**). Conversely, cells in the 25 mg/dL glucose concentration had significantly reduced proliferation rates compared to 100 mg/dL treated cells (**Fig 1**), and the significant decrease (*p* value of < 0.05) did not show until Day 4. Among all three cell lines tested, UM-UC3 cells are most sensitive to glucose fluctuation. At the end of Day 7, UM-UC3 cells cultured in 200 mg/dL glucose demonstrated much higher cell proliferation than HTB-9 and HTB-5 cells, with 16- fold increase compared to 12- and 7- fold in HTB-9 and HTB-5 cells, respectively.

### PKM2 expression in bladder cancer cells

**Fig 2:**
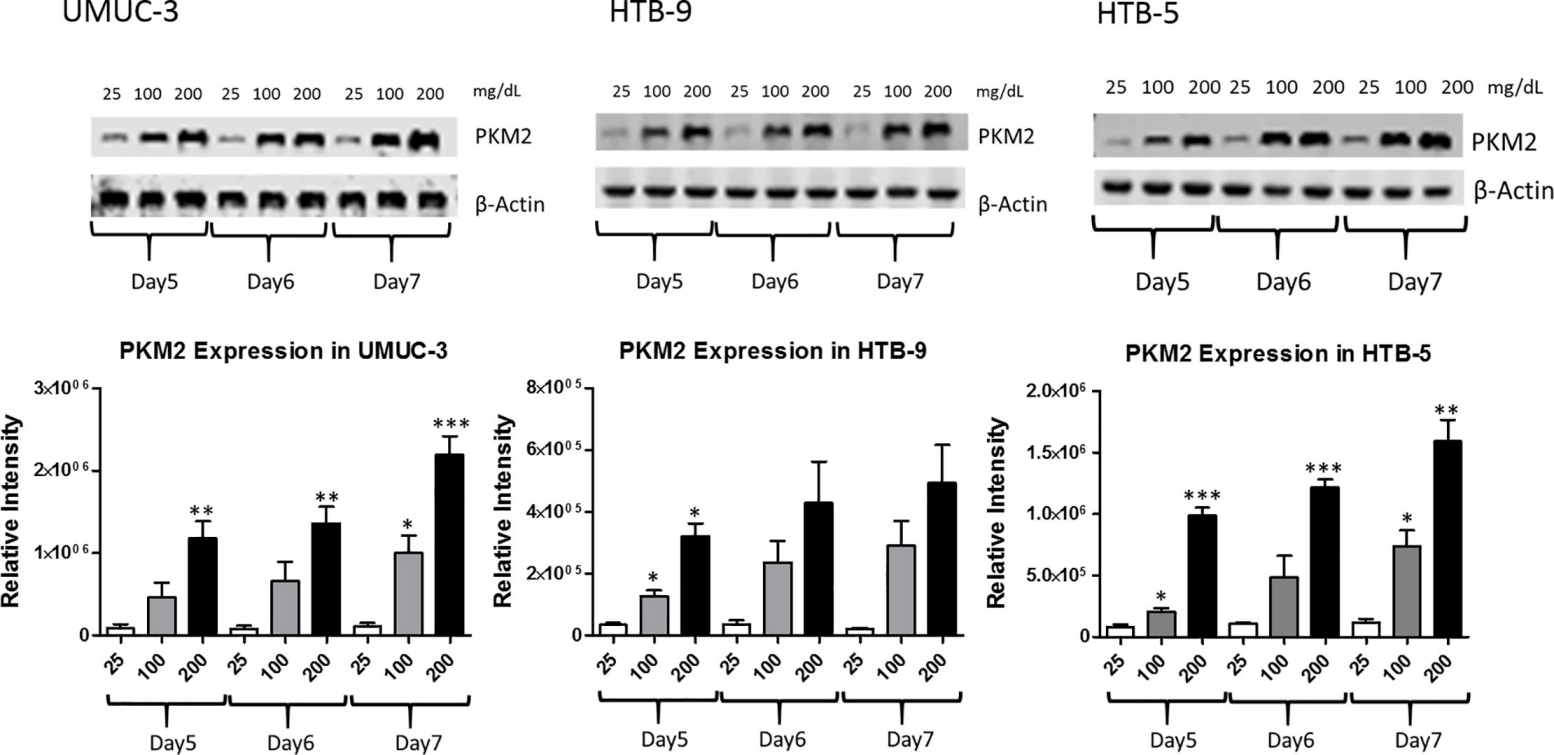
PKM2 expression in bladder cancer cells. UM-UC3, HTB-9, and HTB-5 cells were cultured in media containing different glucose concentrations (25 mg/dL, 100 mg/dL, and 200 mg/dL) for 7 days. On day 5, 6, and 7, protein was extracted and subjected to western blot analysis of PKM2 expression with β-actin as control. Columns represent the mean ± S.E.M. from three independent experiments (n = 3). *, **, and *** indicates significant two-tailed t-test *p* value of < 0.05, < 0.01, and < 0.001, compared with glucose concentration 25 mg/dL at day 5, 6, and 7 respectively.

To evaluate whether PKM2 expression would be elevated in bladder cancer cells cultured under high glucose environment, western blot analysis was carried out in UM-UC3, HTB-9, and HTB-5 cancer cells (**Fig 2**). Briefly, cells were cultured in media containing different glucose concentrations (25 mg/dL, 100 mg/dL, and 200 mg/dL) for 7 days, and the media was changed every 24 hours to maintain glucose levels. On days 5, 6, and 7, protein was extracted and subjected to western blot analysis of PKM2 expression with β- actin as control. On each day of analysis, PKM2 was upregulated at higher glucose concentrations in all the bladder cancer cell lines tested (**Fig 2, upper panel**). Under 100 mg/dL and 200 mg/dL conditions, PKM2 expression level kept rising from day 5 to day 7, which demonstrates increased PKM2 with increased glucose availability (**Fig 2, lower panel**). Western blot analysis matched the cell proliferation data, as increased cell growth in higher glucose concentration correlated with up-regulation of PKM2 protein expression.

### Shikonin inhibits bladder cancer cell growth

**Fig 3:**
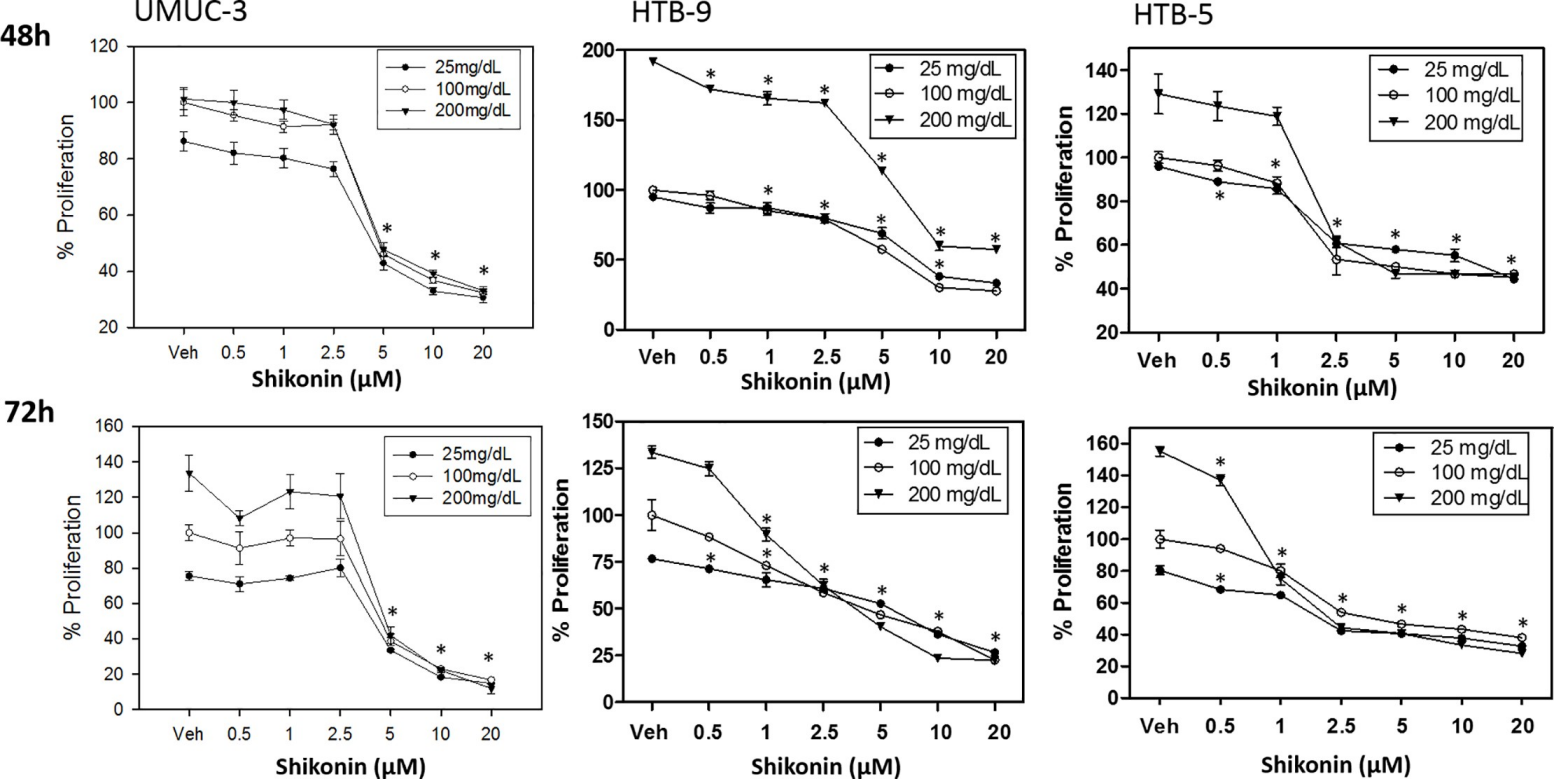
Shikonin inhibits bladder cancer cell proliferation. UM-UC3, HTB-9, and HTB-5 cells were treated with shikonin under three different glucose concentrations 25 mg/dL, 100 mg/dL, and 200 mg/dL. Data were analyzed from three independent experiments, each data point represents the mean ± S.E.M. * indicates significant two-tailed t-test *p* value of < 0.05, compared with vehicle-treated (DMSO) control.

Shikonin, a Chinese herbal drug, has been demonstrated to bind to PKM2 and decrease bladder cancer cell proliferation (T24 cell line) through induction of apoptosis [16]. We utilized shikonin as a PKM2 inhibitor to determine PKM2’s role in glucose metabolism and cellular proliferation in bladder cancer cell lines. UM-UC3, HTB-9, and HTB-5 cells were treated with a range of doses of Shikonin from 0.5 to 20 μM under three different glucose conditions (25 mg/dL, 100 mg/dL, and 200 mg/dL) for 72 hours. Proliferation was assessed by hexosaminidase assay at both 48h and 72h time points (**Fig 3**). UM-UC3 cell line was most responsive to shikonin treatment, 5 μM shikonin inhibited UM-UC3 cell growth around 60% at 48h and 70% at 72h under all three glucose conditions. HTB-9 and HTB-5 cells shared the same trend upon shikonin treatment, but are less sensitive than UM-UC3. Shikonin exposure substantially reduced proliferation of all three bladder cancer cells tested, and the most inhibition of cell growth was observed under highest glucose concentration 200 mg/dL.

**Fig 4:**
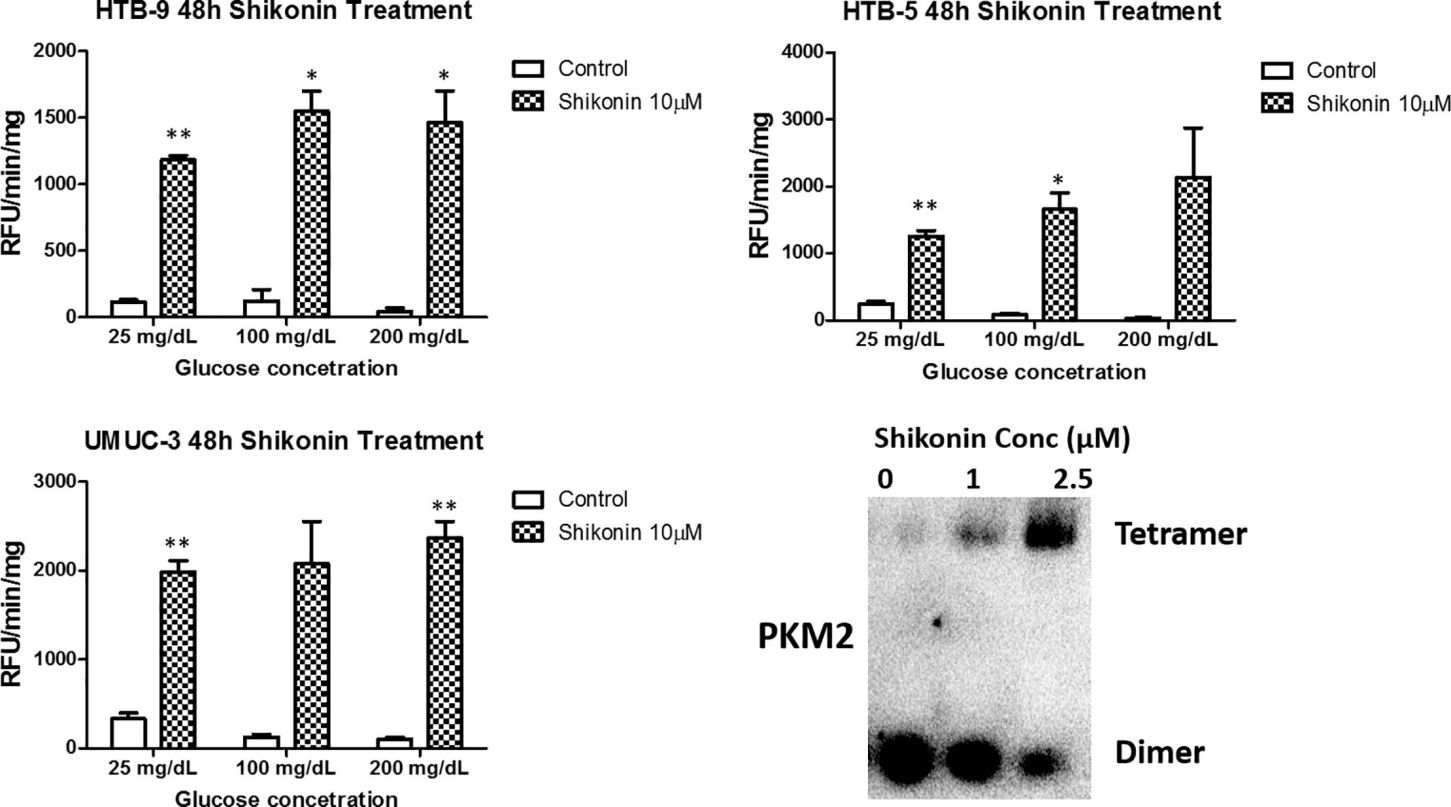
Shikonin induced apoptosis of bladder cancer cells. (A-C): 10μM shikonin treatment over 48h induced activation of Caspase-3 in HTB-9, HTB-5 and UM-UC3 cells. (D): PKM2 tetramer *vs* dimer formation after shikonin exposure for 72h in UM-UC3 cells. Columns depict the mean ± S.E.M. from three independent experiments (n = 3). * and ** indicates significant two-tailed t-test *p* value of < 0.05 and < 0.01 respectively, compared with vehicle-treated (DMSO) control.

Caspase-3 activity was further assessed to measure apoptosis induced by Shikonin. Treatment of UM-UC3, HTB-9 and HTB-5 cells with 10 μM Shikonin for 48 hours resulted in increases in caspase-3 activity, indicating activation of apoptosis by Shikonin (**Fig 4A–4C**). Shikonin was demonstrated to bind PKM and alter PKM2 function, however, the exact mechanism is unclear [10, 17]. Tetramer PKM is a highly catalytic enzyme with high specificity for its substrate and has a high capacity for glycolysis [18]. Dimer PKM has little affinity for PEP, and acts as a brake on glycolysis, funneling intermediates towards other synthetic processes necessary for growth. Given the importance of dimer and tetramer formation of PKM2, we performed Blue Native-PAGE western blot to investigate how Shikonin alters the PKM2 formation in bladder cancer cells. UM-UC3 cell line, based on its high sensitivity to glucose concentration change and Shikonin treatment, was utilized in this study. UM-UC3 cells were treated by two different doses of Shikonin, 1 and 2.5 μM, for 72 hours in 100 mg/ dL glucose media. Blue native studies demonstrated that as Shikonin dosage increased, there was a transition from dimer PKM2 (Tumor M2-PK) to tetramer PKM2 **(Fig 4D)**. As PKM2 dimer and tetramer have fundamentally different enzymatic activities, we suggested the mechanisms of Shikonin in bladder cancer cells may not only act through reductions in glycolysis, but also through alterations in anabolic cancer metabolism that allows for cell growth via synthesis of other necessary intermediates. The changes of PKM2 activity of Shikonin-treated bladder cancer cells, and how Shikonin would regulate the level of PKM2 and PKM1 in the cells will be further investigated in the future, to clarify the mechanisms.

### Urine tumor M2-PK in bladder cancer patients

**Fig 5:**
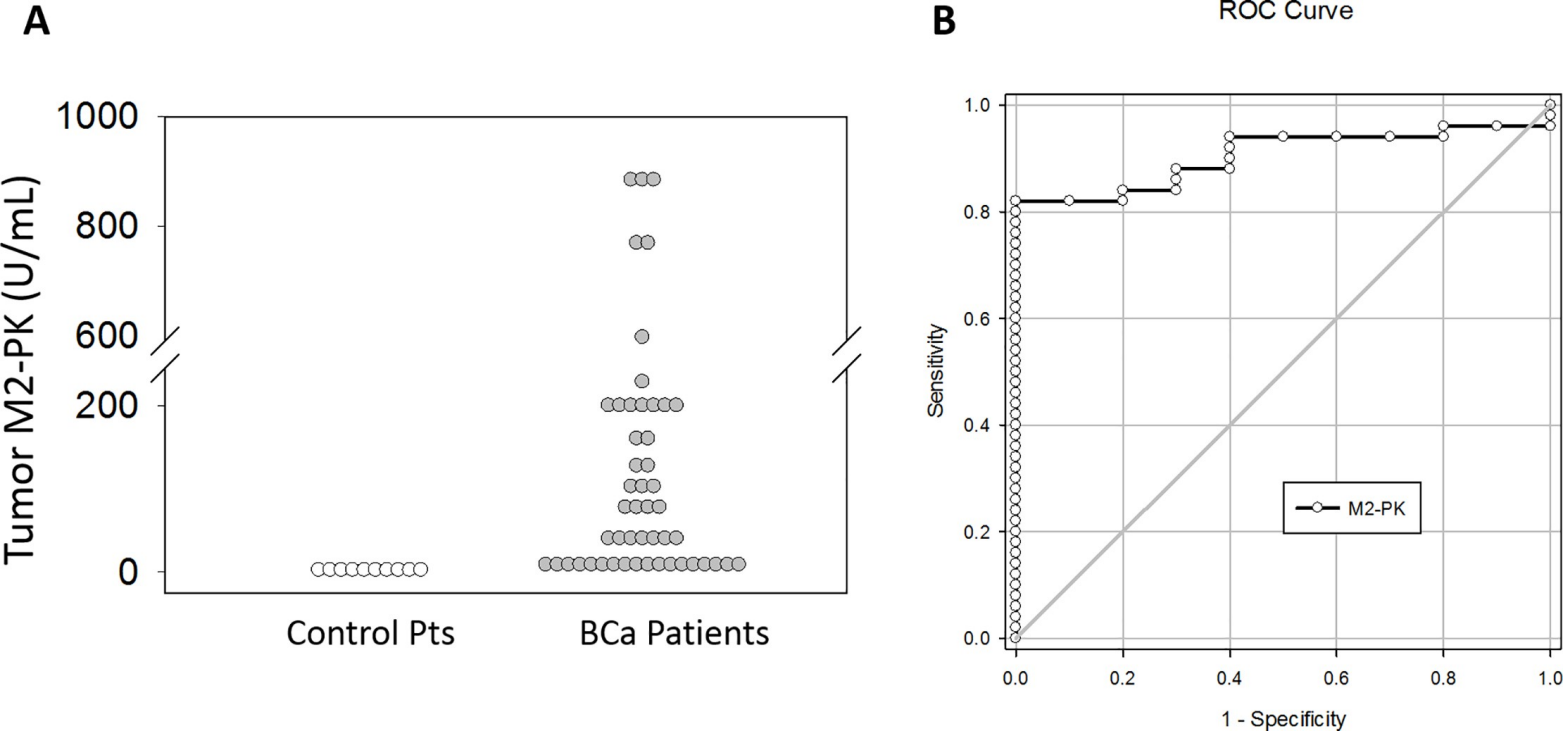
Tumor M2-PK accurately distinguishes controls from patients with bladder cancer. Voided urine was collected from either control volunteers or bladder cancer patients. Tumor M2-PK was performed at various dilutions on urine and M2-PK concentrations were determined (A). Receiver operator characteristic curve analysis was used to assess the capacity of M2-PK to distinguish between control and bladder cancer patients (B).

Given the importance of altered tumor metabolism in tumor development, and the stark contrast previously established in PKM2 expression in normal versus bladder cancer tumors, we wanted to determine if Tumor M2-PK present in urine could serve as a biomarker [19–22]. Fifty urine samples from de-identified bladder cancer patients were obtained from the University of Kansas Medical Center Biorepository and Urology Department Tumor Bank, and ten healthy controls. Characteristics of our study patients with bladder cancer are displayed in Table 1. Urine Tumor M2-PK values were substantially elevated in the majority of patients with bladder cancer (**Fig 5A**).

**Table 1:** Characteristics of the study bladder cancer patients and controls.

	Control (N = 10)
Sex (% Male)	100
Race (% White)	60
Age (years)	43.2
	Bladder Cancer (N = 50)
Sex (% Male)	72
Race (% White)	94
Age (years)	66 ± 5.1
Stage (# of Patients)	
Ta	20
Tis	6
T1	11
T2	10
T3	3
Grade	
High	34
Low	12
Not available	4

Control subjects demonstrated very low levels of Tumor M2-PK in all samples (**Fig 5A**). We used receiver operating characteristic (ROC) curve analysis to determine the capacity of Tumor M2-PK to assess disease (**Fig 5B)**. There was a substantial positive correlation between Tumor M2-PK levels and bladder cancer (area under the ROC = 0.90), and statistical analysis showing 82% sensitivity at 100% specificity using a cut-off of 8.98 units. Moreover, urine Tumor M2-PK test successfully detected several patients with negative/atypical (suspicious) cytology results (**S1 Table**). Compared to cytology, the most commonly used urine-based test for the detection of bladder cancer in both the diagnostic and surveillance settings, which only has a sensitivity of approximately 35% and its role in the diagnostic setting is limited especially in patients with low-grade disease, urinary Tumor M2-PK levels may be a valuable diagnostic marker for the presence of bladder cancer, although further validation is necessary.

## Discussion

The Warburg effect, first described in 1956, is a shift in energy production where cancer cells preferentially produce energy through increase in aerobic glycolysis followed by lactate fermentation, even in the presence of abundant oxygen [2]. A principal driver of aerobic glycolysis, pyruvate kinase, serves as the rate-limiting at last step. The purpose of this study was to further clarify the role of PKM2 in bladder cancer. Pyruvate kinase is predominantly present in its dimer isoform PKM2 in cancer cells, and plays a critical role in tumor growth [7]. PKM2 exists in two forms, a metabolically “active” tetramer, which has a high affinity for PEP and drives energy production, and an “inactive” dimer, which has a low affinity for PEP and allows for substrate accumulation, necessary for cellular proliferation [23]. The oscillation between the tetramer and dimer form of PKM2 is driven in part by the amount of substrate present, including fructose 1, 6 bisphosphate and L-serine. Whereas increased substrate will lead to tetramer formation with subsequent energy production, decreased substrate levels will cause a “switch” back to the dimer form [12, 23]. Despite the fact that bladder cancer is clearly reliant on glucose for proliferation, switching to the active form of PKM2 is paradoxically associated with reduced proliferation. As such, our data support previous hypotheses that activators of PKM2 may block proliferation not through reducing glycolysis, but rather by affecting anabolic metabolism [18].

PKM2 transcripts have been found in 97% of the 131 bladder tumor samples from the TCGA compared to only 3% for PKM1 [11]. PKM2 in bladder cancer has been shown to correlate with increasing grade of tumor tissue [9]. As such, bladder cancer clearly expresses significantly more PKM2 compared to PKM1, which may provide survival advantages. Our studies further characterize the importance of PKM2 in bladder cancer. We demonstrate that PKM2 is upregulated in conditions where increased glucose is available for bladder cancer cells and correlates with increased proliferation. The bladder cancer cells may need 24–48 hrs to adapt to the increased/decreased glucose dependent on different cell lines, but the significant change in proliferation is detected as early as 48 hrs for HTB-9 cells. The longer the cells adjusted to the higher glucose level, the more upregulated PKM2 levels were observed across all the bladder cancer cell lines. Additionally, we found that Shikonin, a known PKM2 inhibitor promotes a shift of PKM2 from the inactive dimer to the tetramer form correlating with decreased cellular proliferation with increasing drug dose. Shikonin has been shown to kill certain cancer cells (include bladder cancer cells) and inhibited cells migration and invasion through many possible mechanisms, including the inhibition of protein tyrosine kinase, the DNA topoisomerases activities, and tumor necrosis factor receptor TRAP1 expression [16, 17, 18, 24]. Other mechanisms involved in shikonin-induced cancer cell death include upregulation of P53. Even our data show shikonin can cause bladder cancer cell death at certain dose, and plays an important role in PKM2 dimer/tetramer conversion, the exact mechanism of PKM2-specific effects by shikonin remains incompletely understood, and requires further investigation. To further clarify the mechanism behind, in our next study, the PKM2 activity of all shikonin-treated bladder cancer cells will be measured, together with the expression level of PKM1, which might be upregulated during PKM2 inhibition and reduce cancer cell proliferation.

The important role PKM2 played in bladder cancer led us to evaluate the possibility of Tumor M2-PK as a urine-based biomarker for the detection of bladder cancer. Currently, cytology is the most commonly used urine-based test for the detection of bladder cancer in both the diagnostic and surveillance settings. However, cytology has a sensitivity of approximately 35% and its role in the diagnostic setting is limited especially in patients with low-grade disease [25]. Other urine-based markers including BTA, ImmunoCyt, and NMP22, have not demonstrated enough accuracy to warrant inclusion in the AUA guidelines on asymptomatic hematuria or treatment of non-muscle invasive bladder cancer [26, 27]. Fifty bladder cancer patients and ten normal controls were measured, and Tumor M2-PK values were elevated in the urine of patients with bladder tumors with statistical analysis showing 82% sensitivity. Notably, levels in multiple patients were more than two orders of magnitude above control values, and this urine Tumor M2-PK test successfully detected 10% patients within the whole group, who were falsely diagnosed with negative/atypical (suspicious) cytology results. Tumor M2-PK has been found to be a significant tumor marker both in serum and feces for renal cell carcinoma, pancreatic cancer, and colorectal cancers [19, 12, 28]. To our knowledge, this is the first report in utilizing Tumor M2-PK as a urinary marker of disease. Considerable elevation in Tumor M2-PK values, 82% assay sensitivity, combined with the fact that urine is readily assessable from patients may yield a valuable initial marker for clinicians that are suspicious of bladder cancer. Typically, the earliest sign of bladder cancer is hematuria; however, this is not a specific finding and the vast majority of patients do not have a malignant finding afterwards, which leads to unnecessary instrumentation with cystoscopy and imaging. In fact, in a study of 2,640 patients, only 55 (2.1%) were found to have a bladder tumor [25]. A point of urine test for Tumor M2-PK may yield a rapid, valuable assay for clinicians to assess potential course of patient treatment. In a small clinic trial of patients with diabetes mellitus and bladder cancer, Tumor M2-PK levels returned to normal after bladder tumor resection (**S1 Fig**). Due to the limited pool of current patients, we do not find direct connection between PKM2 levels and the grade and stage of bladder cancer patients in this study. Future studies will aim at enrolling more patients with more diversified ages, race and sex to perform the analysis on grade and stage association of PKM2 levels in bladder cancer. Our future study also aims at understanding the mechanisms behind how glucose level change can affect PKM2’s function in bladder cancer.

## Conclusions

We provide qualitative and quantitative information about the role PKM2 plays in bladder cancer. Bladder cancers demonstrated elevated levels of PKM2, a principal driver of the Warburg effect. In addition, Tumor M2-PK values were elevated in the urine of patients with bladder tumors, and the statistical analysis showed 82% sensitivity of the test. PKM2, specifically the dimer (Tumor M2-PK) has the potential for serving as both a urinary biomarker as well as a target of drug therapy in bladder cancer.

## Supporting information

S1 Fig Urine tumor M2-PK values for bladder cancer patients with diabetes (before and after surgery). Urine Tumor M2-PK values were determined for bladder cancer patients with diabetes, values shown including both before and after treatment. Most patients’ Tumor M2-PK levels returned to normal after the tumor was removed. Note: Patient 3 dropped off the trial after first visit.
(DOCX)

S1 Table Characteristics of the study bladder cancer patients. (XLS)
