# Supplementary material for: Tumor M2-PK: A novel urine marker of bladder cancer
Source: PLoS One. 2019 Jun 27;14(6):e0218737. doi: 10.1371/journal.pone.0218737 (PMC6597081; doi:10.1371/journal.pone.0218737)
Supplement: S1 Fig — Urine Tumor M2-PK values were determined for bladder cancer patients with diabetes, values shown including both before and after treatment. Most patients’ Tumor M2-PK levels returned to normal after the tumor was removed. Note: Patient 3 dropped off the trial after first visit. (DOCX) [file pone.0218737.s001.docx]

**S2 Figure. Urine Tumor M2-PK values for bladder cancer patients with diabetes (before and after surgery)**


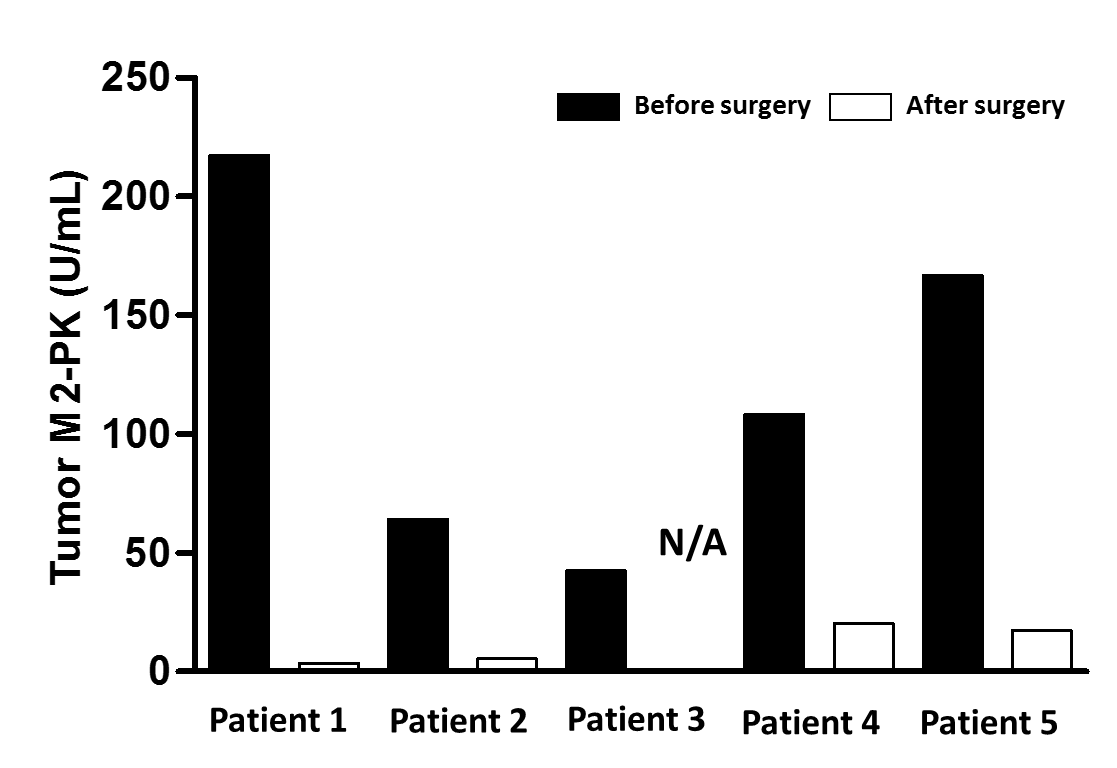


**S2 Fig.** Urine Tumor M2-PK values were determined for bladder cancer patients with diabetes, values shown including both before and after treatment. Most patients’ Tumor M2-PK levels returned to normal after the tumor was removed. Note: Patient 3 dropped off the trial after first visit.
